# Epidemiological Review of Francisella Tularensis: A Case Study in the Complications of Dual Diagnoses

**DOI:** 10.1371/currents.outbreaks.8eb0b55f377abc2d250314bbb8fc9d6d

**Published:** 2018-01-18

**Authors:** Ralph Anthony Stidham, David B. Freeman, Robert L. von Tersch, Peter J. Sullivan, Samantha D. Tostenson

**Affiliations:** Epidemiology and Disease Surveillance, US Army Public Health Command-Central, JBSA Fort Sam Houston, Texas, United States of America; † Laboratory, Diagnostics Section, US Army Public Health Command-Central, JBSA Fort Sam Houston, Texas, United States of America; † Office of the Commander (Commander), US Army Public Health Command-Central, JBSA Fort Sam Houston, Texas, United States of America; Veterinarian Services, Fort Riley Veterinary Treatment Facility, Fort Riley, Kansas, United States of America; Special Pathogens Laboratory, United States Army Medical Research Institute of Infectious Diseases (USAMRIID), Fort Detrick, Maryland, United States of America

## Abstract

**Introduction:**

Tularemia is a rare but potentially fatal disease that develops in numerous wild and domestic animals, including lagomorphs, rodents, cats, and humans.  Francisella tularensis bacterium, the causative agent of tularemia, was identified by veterinary personnel at Fort Riley, Kansas during a routine post-mortum evaluation of a domestic feline. However, before formal diagnosis was confirmed, the sample was sent and prepared for rabies testing at the Department of Defense (DoD) U.S. Army Public Health Command Central (PHC-C), Food Analysis and Diagnostic Laboratory (FADL).  This case report provides insight on how veterinarian staff and laboratory personnel can clinically manage esoteric, unexplained, or post-mortum examinations.  The epidemiologic characteristics of tularemia, F. tularensis as an organism of military interest, potential laboratory management of F. tularensis, and clinical findings on a case of feline tularemia are discussed. It further raises questions as to whether or not dead animals should be treated as sentinels and be pre-screened for select agents, especially in instances of dual diagnoses.

**Methods:**

A necropsy was performed on the cat by the Fort Riley veterinarian, DNA extraction and PCR analyses were conducted by FADL microbiologists, histology and immunohistology analyses were conducted by the Kansas State Veterinary Diagnostic Laboratory, and feline tissue and blood were sent to the U.S. Army Medical Research Institute of Infectious Diseases (USAMRIID) for confirmatory testing and strain identification of tularemia.

**Results:**

Tularemia was identified in the spleen of the cat by the Fort Riley veterinarian and during the histological sampling of the spleen by the Kansas State Veterinary Diagnostic Laboratory.  A specific subsequent real-time polymerase chain reaction (RT-PCR) in vitro diagnostic detection of target DNA sequences of F. tularensis was conducted by the FADL microbiologists using a Joint Biological Agent Identification and Diagnostic System (JBAIDS) Tularemia Detection Kit to detect a presumptive qualitative result to detect tularemia in feline and blood samples.   USAMRIID also performed RT-PCR and identified genomic DNA from F. tularensis Type A, (SPL15.013.02), thus confirming the FADL’s initial presumptive result of F. tularensis.  USAMRIID attempted to culture F. tularensis from three samples (swab, feline tissue, and transfer pipette tip), but no growth consistent with F. tularensis was observed on the cysteine heart agar with sheep blood and antibiotics (CHAB) and chocolate (CHOC) plates.

**Discussions:**

Our case study of a dual diagnosis of presumptive F. tularensis and possible rabies exposure transmission from a pet cat to its owner provides insight on how veterinarian staff and laboratory personnel can clinically manage esoteric, unexplained, or post-mortum examinations.  Our case study also demonstrates the obligation for cooperation between animal health, human health, and public health professionals in the management of zoonotic diseases.

## Introduction

“The causative agent of tularemia,* Francisella tularensis*, is one of the most infectious pathogenic bacteria known….” (Dennis et al^1^.)

The proximity of household pets and the current view that pets are "family members" within households^2^ place pet owners and the general public increasingly at risk for exposure to various zoonotic diseases. Cats, in particular, are responsible for the transmission of an extensive array of infectious pathogens^3^. Herein, the significant clinical and epidemiologic characteristics of tularemia are described in a domestic cat highlighting essential underpinnings of potential* F. tularensis* laboratory exposure management and the protective role veterinarians and laboratory personnel assume for public health safety. Furthermore, this case study raises questions as to whether or not dead animals should be treated as sentinels and be pre-screened for select agents, especially in instances of potential dual diagnoses.

**Epidemiology: Human Exposure and Geographic Distribution**

Tularemia is a particularly adaptive zoonotic disease caused by a small, non-motile, aerobic and fastidious gram-negative pleomorphic coccobacillus bacterium, *F. tularensis*. Colloquial names for this condition include Francis' disease, deer-fly fever, rabbit fever, water-rat trappers' disease, wild hare disease (yato-byo), and Ohara's disease^4^. The organism is named for Edward Francis, a US Public Health surgeon who dedicated his life to researching the organism; and for Tulare County, California, where the syndrome was first described in ground squirrels in 1911^5^. This organism can infect humans and a diverse population of animals, including more than 200 species of wild and domestic mammals^5^^,^^6^^,^^7^^,^^8^. Following an incubation period of 3 to 5 days (range, 1 to 21)^4^, infection with *F. tularensis* can result in various clinical presentations, depending on the route of inoculation, the dose of the inoculum, and the virulence of the organism. Humans become infected by various methods, including arthropod bites, which exemplifies a main route of contamination, handling infected animal tissues or fluids, direct contact with or ingestion of contaminated water, food, or soil, and inhalation of infective aerosols (e.g. intentional or unintentional aerosolization)^1^^,^^9^. While individuals of all ages and both sexes appear to be equally susceptible to tularemia, selected activities such as hunting, trapping, butchering, and farming are most likely to expose adult men^1^. Although * F. tularensis* is extremely infectious and pathogenic, its transmission from person to person has not been recorded^4^^,^^5^.

Tularemia is primarily a disease of wild lagomorphs (rabbits and hares) and rodents in the Northern Hemisphere. Albeit from older research, there is no paucity of reports^5^^,^^8^^,^^10^^,^^11^^,^^12^^,^^13^^,^^14^^,^^15^ demonstrating the occurrence of cases of tularemia through contact with infected cats. In fact, more than 50 human cases of tularemia following cat bites were reported between 1928 and 1993^13^. Cats characteristically acquire tularemia through predation of infected lagomorphs and rodents or from bites by ticks that have ingested blood from an animal^8^^,^^16^. Cats can directly (bites and scratches) and indirectly (transfer of infected ticks) transmit tularemia to humans despite not exhibiting any signs of clinical illness. Virtually all of the documented cases of tularemia in domestic cats^8^^,^^10^^,^^11^^,^^12^^,^^13^^,^^14^^,^^15^ and those involving transmission of this disease from cats to humans have two associated factors: 1) the domestic cats are permitted to roam outside of the home; and 2) they hunt, kill, and consume infected small rodents and rabbits. Therefore, the risk of acquiring tularemia infection from domestic cats is not negligible for pet owners and by extension veterinarians, veterinary technicians, and laboratory personnel analyzing samples from such animals.

*F. tularensis* has been reported in humans and animals from every state except Hawaii^17^. Approximately 125 cases have been reported annually in the United States during the last two decades^17^. The number of tularemia cases reported annually in the United States has diminished significantly from approximately 2000 cases since the first part of the 20th century to a current average of 125 cases the last two decades^17^. The incidence was highest in 1939, when 2,291 cases were reported^6^ and remained high throughout the 1940s. The number of cases drastically reduced in the 1950s and 1960s to the comparatively constant number of cases reported since that time^6^. There are no specific data available that explain the decline in cases since the 1950s. However, it is assumed that this may be due to less-frequent exposure of humans to rodents, rabbits and hares which in turn may be related to a decrease in the number of hunters and a decrease in the percentage of the population living in rural settings^18^.

Between the years 2001-2010, Kansas (where Fort Riley is located), along with 6 other states in the south-central United States, to include highly endemic areas in Arkansas, Oklahoma and Missouri, reported 59 percent of the total 1,208 reported cases of tularemia^17^. Currently, almost all cases of tularemia customarily still occur in the south-central states. To illustrate this, over half of the reported cases in 2013 were in Arkansas, Missouri, Kansas, Nebraska, and Oklahoma^19^. Correspondingly, the number of reported cases in Colorado, Nebraska, South Dakota, and Wyoming increased considerably in 2015^20^. The events described in our case occurred in the neighboring state of Kansas and was disclosed to the CDC concurrent to the events as they unfolded^20^.

The exact reasons for the incidence increase are unknown but are most likely associated with the complex ecology of the disease and the complicated relationship between arthropods (ticks or deer flies), rodent or rabbits, and human activity. These factors are further influenced by environmental factors such as increased rainfall which can fuel vegetation growth^20^ and the accompanying increase in rodent populations can complicate predator population dynamics, along with unpredictable opportunities for spillover into human activity such as hunting and trapping. An illustration of this is that most cases now result from arthropod bites during the summer months (between May through August), whereas a winter peak in incidence cases mostly occur among hunters and trappers who handle infected rabbits^7^ and other animal carcasses.

Enhanced disease surveillance cognizance by state public health departments and a heightened clinical suspicion to test for tularemia by clinicians and veterinarians could also be additional explanations. Even with these numbers reported each year, cases of tularemia in humans in the United States are unquestionably being unrecognized and underreported by animal and human health professionals alike^21^. Perhaps a portion of patients in whom community-acquired pneumonia is diagnosed but the etiologic agent is not recognized essentially have tularemia and are spontaneously and fortunately cured by the unanticipated choice of fluoroquinolones or aminoglycoside antibiotics as a treatment^22^.

When tularemia cases in the United States are reported every year, the typical pattern involves small clusters, most often sporadic human cases. The cases tend to involve a rabbit enzootic cycle, ixodid tick vectors, and *F. tularensis* subspecies *tularensis* genotype A1^23^ (see below), likely to occur in males more so than females, in children younger than 10, and in adults >50 years of age or older^1^,and cases may exhibit human activity patterns that increase opportunities for disease exposure. A clear example of this pathogen’s epidemic potential was exhibited in Europe through outbreaks on the Soviet and German eastern front during WWII as well as various epizootic-associated outbreaks that previously occurred in the United States^1^.

**Taxonomy of *F. tularensis***

While there are taxonomy analysis and strain variations, formulations of a number of distinctive biochemical, epidemiological, virulence, and pathogenesis data have been used to divide *F. tularensis* into 4 subspecies, two of which (type A and B) cause disease in cats^5^^,^^24^^,^^25^. Tularemia can be observed as being caused by type A, and type B subspecies, which distinguishes the highly virulent strains of the bacterium (type A) for humans^1^ from less virulent strains (type B). *F. tularensis* subsp. *tularensis* (Type A) is the most common type in North America, associated with a tick-rabbit cycle^24^. Jellison type B (*F. tularensis* subsp. *holarctica*, and previously subsp. *palaearctica*)^8^^,^^25^, is a less virulent type, responsible for human tularemia infection in the Northern Hemisphere, to include Europe and Asia as well as North America.

**Potential Use in Bioterrorism**

Due to its status as one of the most infectious pathogenic bacteria known, requiring inoculation or inhalation of as few as 10 organisms to initiate human infection, *F. tularensis* continues to be an organism of military interest^1^. *F. tularensis* is considered to be a dangerous biological weapon because of its ability to infect via aerosol, severe infectivity, history of being developed as a bioweapon, and capability for considerable harmful medical sequelae to include illness and death^1^. Respiratory or pneumonic tularemia following intentional release of a virulent strain of *F. tularensis* for nefarious purposes would have a tremendous, devastating impact and cause high morbidity and mortality in large population groups^1^^,^^26^^,^^27^^,^^28^. For these reasons, *F. tularensis* is a nationally reportable disease by civilian^29^ and military^30^ veterinarians and other public health professionals. Because of their contact with animals and increased awareness of zoonotic diseases, veterinarians are in the unique position of being able to alert medical personnel to the possibility of exposure to *F. tularensis* and to promptly report the disease to local public health authorities. Our case with this organism demonstrates the close linkage that should exist between military and civilian public health, infectious disease professionals, and law enforcement charged with protecting the public. Consequently, the Federal Bureau of Investigation became briefly engaged in this situation and worked with the Army PHC-C Commander (COL von Tersch, personal communication) to identify this case as an epizootic event as opposed to a bioterrorism occurrence.

**Laboratory Management of F. tularensis**

Prior to the availability of live vaccines, laboratory-acquired infections with *F. tularensis* in laboratory personnel was a frequent occurrence, especially in labs investigating infectious agents^31^^,^^32^. However, modern understanding of tularemia and implementation of biosafety rules and regulations drastically minimized the risk of accidental exposure, but unfortunately the threat remains^33^^,^^34^. The most efficient methods of managing the problem of accidental laboratory infections is to prevent their occurrence through a combination of engineering controls, training in proper lab techniques, employee vaccination, and an active biosafety program^33^^,^^34^. Should an exposure happen despite these efforts, a laboratory must have standard practices in place to promptly deal with any consequence in order to ameliorate any impacts to the exposed individual in the laboratory environment.

Laboratory persons working with infectious agents or potentially infected materials must be aware of exposure hazards, and be trained and proficient in the standard practices and techniques required for handling such material safely. Particularly, *F. tularensis* is highly infectious when grown in culture, and laboratory-acquired infections have been documented^31^^,^^32^^,^^35^^,^^36^^,^^37^. Standard Biosafety Level (BSL) 2 practices, containment equipment, and facilities are recommended for activities involving clinical materials of human or animal origin suspected or known to contain *F. tularensis*^33^^,^^34^. For example, according to the American Society for Microbiology, human clinical specimens suspected of containing *F. tularensis* can be processed with BSL-2 practices^34^. Laboratory personnel should be notified of the possibility of tularemia as a differential diagnosis when samples are submitted for diagnostic tests. BSL-3 and ABSL-3 (A, Animal) practices, containment equipment, and facilities are recommended for all handling of suspect cultures and isolates, animal necropsies, and for experimental animal studies. Preparatory work on cultures or contaminated materials for automated identification systems should be performed at BSL-3^33^.

When collecting or handling clinical specimens, laboratory personnel should 1) handle all specimens in a BSL-2 laminar flow hood with protective eyewear (e.g., safety glasses or eye shields,); 2) use closed-front laboratory coats with cuffed sleeves, and stretch the gloves over the cuffed sleeves; 3) avoid any activity that places persons at risk for infectious exposure, especially activities that might create aerosols or droplet dispersal; 4) decontaminate laboratory benches and equipment after each use and dispose of contaminated supplies and equipment (as appropriate) in proper receptacles; 5) wearing gloves to protect from infections through the skin; 6) remove and reverse their gloves before leaving the laboratory and dispose of them in a biohazard container, and wash their hands and remove their laboratory coat^34^^,^^38^.

**Managing Animal Necropsy**

Those involved in animal necropsy practice have a duty not only to ensure that they are aware of the hazards and risks associated with such work, but also to take steps to minimize these risks. The hazards present within an animal body are often unknown at the start of the necropsy. Fastidious preparation in all cases, and not just those where a risk has been identified, is crucial. Veterinarian staff and laboratory personnel must clinically manage esoteric, unexplained, or questionable pet death examination as if the animal were contaminated and follow veterinary standard precautions as noted in the Compendium of Veterinary Standard Precautions for Zoonotic Disease Prevention in Veterinary Personnel^39^ and the Infection Control and Biosecurity Standard Operating Procedures by the James L. Voss Veterinary Teaching Hospital^40^. This would involve methodical hand hygiene procedures that include handwashing with soap and water, the use of alcohol-based hand rubs, and the proper use of gloves; facial protection such as the use of a N95 respirator, surgical mask, or a face shield or goggles worn with a surgical mask during procedures that cause potential infectious sprays and splashes and the use of protective outerwear such as laboratory coats, aprons, coveralls, nonsterile gowns, and footwear^39^^,^^40^. It would be optimal if necropsies in private veterinary clinics would use appropriate personal protective equipment (PPE) such as gloves and face protection, use negative airflow rooms, downdraft necropsy tables, and apply respiratory precautions. Civilian veterinarians may need to defer necropsies to facilities such as university research facilities in order to have negative airflow rooms and downdraft necropsy tables; facilities such as the Holland Military Working Dog (MWD) Hospital at Joint Base San Antonio (JBSA) – Lackland Air Force Base in San Antonio, Texas and the MWD Center at Fort Sam Houston, Texas have negative airflow rooms and downdraft necropsy tables for military veterinarians (LTC Douglas Owens, personal communication).

**Case Report**

A 7-year-old indoor-outdoor male domestic shorthair cat was exhibiting atypical behavior as described by the owner and was identified as visibly ill. When the owner attempted to collect the cat, it subsequently bit the owner, escaping underneath a bed. The owner found the cat dead several hours later without any visible signs of trauma or obvious cause. The expired cat was brought to the Irwin Army Community Hospital emergency room (Kansas) during medical analyses of the bitten owner and collected the next day by personnel from the Fort Riley Veterinarian Treatment Facility (VTF), where a necropsy was performed. The cat was never presented as a patient at the VTF, so no medical record had ever been generated for the animal. The vaccination status of the cat was unknown. In the interest of the owner’s health, and abiding by all regulatory requirements^33^, the attending veterinarian disarticulated the animal’s head and sent it to the FADL at JBSA-Fort Sam Houston for rabies diagnostic testing. While the head was in transit, the veterinarian conducted a necropsy and discovered nodes on the cat’s spleen which raised concern for potential infection for F. tularensis. The FADL was notified that same evening via e-mail citing preliminary histopathological concerns for tularemia infection; however the e-mail was not read until the following afternoon after the veterinarian called the FADL. Upon arrival at the FADL the next day, the sample was handled according to standard operating procedures and placed in the immunodiagnostic laboratory. The FADL personnel were unaware of the potential tularemia-positive sample until the Ft Riley veterinarian called and identified the possibility. Once the sample was discovered to be atypical, the FADL molecular diagnostic section conducted quick searches as to the risks associated with *F. tularensis* while prepping the laboratory to test for the primary request, rabies. The submitted sample (cat head) still remained in the packaged box. At this time, the FADL personnel contacted the Army Medical Department Center and School (AMEDDC&S) to obtain an FDA-approved *F. tularensis* kit for diagnostic testing. Blood and small tissue samples were carefully removed under BSL-2 conditions with proper PPE and transported to the molecular diagnostic section and tested for* F. tularensis*.

## Materials and Methods

**DNA extraction and PCR analyses (FADL)**

Consent was obtained from the cat's owner to be used in this research study. DNA was extracted and isolated from approximately 50 µL of sequestered cat blood via a DNeasy Blood & Tissue Kit (Qiagen, Inc., Hilden, Germany) by FADL microbiologists. Subsequent real-time polymerase chain reaction (RT-PCR) in vitro diagnostic detection of target DNA sequences of *F. tularensis* was conducted to obtain a qualitative result using a Joint Biological Agent Identification and Diagnostic System (JBAIDS) Tularemia Detection Kit (Idaho Technology, Inc. [ITI], Salt Lake City, Utah) that detects a diverse panel of 27 *F. tularensis* strains. Each sample batch contained water as a negative control. DNA concentration and purity were determined with the Nanodrop ND-1000 spectrophotometer (Thermo Scientific, Wilmington, DE, USA). To verify the presumptive positive result, feline tissue and blood were sent to the U.S. Army Medical Research Institute of Infectious Diseases (USAMRIID) for confirmatory testing and strain identification.

**Histology and Immunohistology (Kansas State Veterinary Diagnostic Laboratory, KSVDL)**

Formalin-fixed tissue specimens from the cat were embedded in paraffin, sectioned at 4 um, and stained with Hematoxylin and Eosin (HE).

**Bacteriology (USAMRIID)**

Samples obtained from the feline from the Fort Riley VTF were sent and referred for *F. tularensis* testing by the FADL. USAMRIID received a blood covered swab, approximately 1.0 gram of unknown tissue and a micropipette tip with blood residue.

The blood covered swab was streaked on cysteine heart agar with sheep blood and antibiotics (CHAB) and chocolate (CHOC) plates. The swab was then placed in 500 µl Dulbecco's phosphate-buffered saline (DPBS) and vortexed for PCR extraction. Following the kit manufacturer instructions, the Qiagen QiaAmp DNA Mini Kit was used to extract the DPBS.

The tissue was broken up with a 1 µl sterile loop which was used to streak CHAB and CHOC plates. The Qiagen QiaAmp DNA Mini Kit was used to extract 0.10 grams of tissue following kit manufacturer instructions for DNA purification from tissues.

The pipette tip was swabbed with a DPBS moistened sterile swab and streaked on CHAB and CHOC plates. Extraction was not performed on the pipette tip material.

The culture plates were incubated at 37°C ambient atmosphere for 8 days and observed daily for characteristic *F. tularensis* growth

Each extract was run on the Applied Biosystems 7500 Fast Dx Real-Time PCR instrument using Invitrogen Platinum Taq and the C- reactive protein (CRP) Real-Time PCR assay for detection of *F. tularensis*. The following run conditions were used:

**Table:** Detector: FAM Quencher:None Passive Reference: None Run Mode: Fast 7500 Sample Volume 20 ul

Step	Cycles	Temp	Time
Denature	1	50C	2 min
Hot Start	1	95C	2 min
PCR	45	95C	3 sec
		60C	30 sec

## Results

**Clinical and Gross Findings (Ft Riley Veterinarian)**

The remains of the cat were picked up from Irwin Army Community Hospital emergency room by Fort Riley VTF personnel and brought back to their facility for a post-mortem examination. Findings on the initial physical examination of the deceased cat were unremarkable. On necropsy, no superficial trauma was noted. Widely distributed copious pinhead sized miliary white necrotic foci along with relatively small spheroidal lesions that were uniformly white or yellow in appearance were noted throughout the conspicuously enlarged splenic capsule and cut surface (Figure 1).

**Tularemia Spleen From Gross Necropsy 2:**
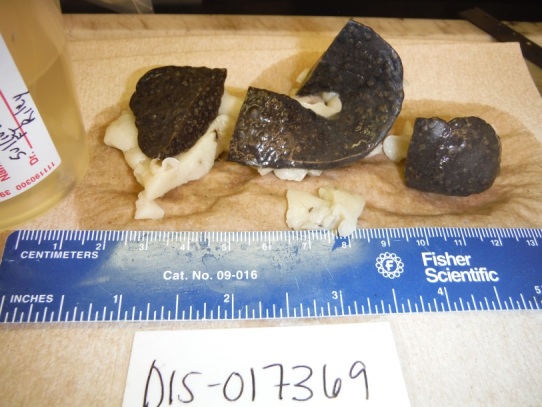
Photograph of a spleen obtained from the gross necropsy of a 7 year old cat after fixation. Reprinted under a CC by license, with permission from Dr. Kelli Almes, original copyright 2017.

The post-mortem examination was immediately terminated after the spleen was examined and removed for testing.

**Histopathological Findings (KSVDL)**

The Kansas State pathologist performed histological sampling of the spleen with Hematoxylin and Eosin stains. Typical macroscopic lesions were observed and consistent with those typically seen in cats with *F. tularensis*. The spleen showed multifocal, individual to coalescing foci of inflammation and necrosis mainly situated upon and eroding the white pulp. The foci were somewhat differentiated from adjacent red pulp. Throughout the splenic parenchyma, there were extensive multifocal to coalescing areas of inflammation and necrosis characterized by infiltration of large numbers of an admixture of intact and degenerate neutrophils and macrophages admixed with conspicuous cellular debris and fibrin. Immunohistochemistry stain for *F. tularensis* was positive (Figure 2).

**A photomicrograph of immunohistochemistry for Francisella tularensis.**
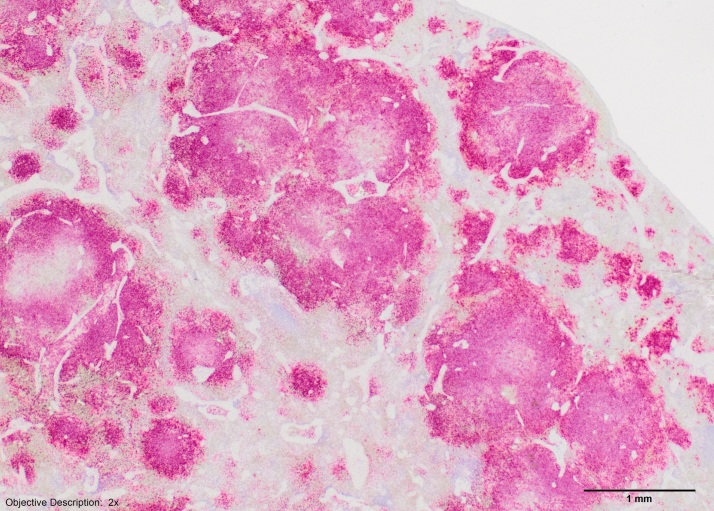
Note the large amount of positive staining (red) throughout the tissue that corresponds to the gross lesions observed (Figure 1). Reprinted under a CC by license, with permission from Dr. Kelli Almes, original copyright 2017.

**Molecular Analysis (FADL)**

After receiving the cat head from the Fort Riley VTF, DNA extraction from blood and tissue samples were performed. Subsequent PCR testing conducted with a JBAIDS Tularemia Detection Kit showed an amplification curve with a crossing point (CP) of 27.55, indicating a presumptive positive result. Typical *F. tularensis* positive blood culture samples amplify on the tularemia assay test with CP values ranging from 24.86 to 29.78.

**Bacteriology Findings (USAMRIID)**

To verify the presumptive positive result, small tissue samples (feline sternohyoid and peripheral connective tissue) were excised from the cat’s head and sent to the U.S. Army Medical Research Institute of Infectious Diseases (USAMRIID) for confirmatory testing and phenotyping. Upon receipt, USAMRIID followed CDC guidelines for definitive tularemia diagnosis by attempting to culture *F. tularensis* from three samples (swab, feline tissue, and transfer pipette tip). Unfortunately, no growth consistent with *F. tularensis* was observed on the chocolate or the CHAB plates, partially attributed to mixed background flora. However, USAMRIID also performed RT-PCR and identified genomic DNA from* F. tularensis* Type A, (SPL15.013.02), thus confirming our initial presumptive result of *F. tularensis*.

**Discussion**

This case study illustrated not only the veracity of the maxim “chance favors the prepared mind” exquisitely because of the judicious, albeit auspicious, identification of the assiduous bacterium by the owner’s veterinarian, but also the high level of patient care that can potentially be achieved when veterinary personnel actively foster working relationships with their local public health counterparts. In this case, the owner was prophylactically treated with doxycycline for* F. tularensis* exposure within 24 hours of being bitten and given the rabies post-exposure prophylaxis (human rabies immune globulin [HRIG] and the rabies vaccine) the same day of being bitten. This is the good news.

On the other hand, the FADL immunodiagnostic personnel were not prepared and were unaware of the potential tularemia-positive sample up until the veterinarian called and identified the possibility the next day. As a result, four FADL laboratory personnel were potentially exposed to the positive sample while handling it under standard operating procedures. As a precautionary measure, the four FADL personnel initially associated with the tularemia cat head were sent to Occupational Health at San Antonio Military Medical Center. Fortunately, each individual that was examined by an infectious disease physician were deemed to be low risk (minimal exposure risk due to the laboratory personnel using appropriate gloves, surgical masks, nonsterile gowns, and handling the sample within a biological safety cabinet) for developing tularemia and were given a prescription for doxycycline (100mg twice daily for 14 days), a tularemia post-exposure prophylaxis.

Once the sample was discovered to be atypical, FADL personnel conducted quick internet searches as to the risks associated with *F. tularensis* while prepping the laboratory to test for the primary request, rabies. Overall, better preparation in handling an anomalous sample would have resulted in testing this cat in the appropriate setting to decipher an accurate dual diagnosis. Recurrent phone calls, including a joint conference call, ensued with various divisions of the CDC - the Rabies Laboratory at the CDC’s Pox and Rabies Branch (PRB) in Atlanta, which performs rabies testing, and the Division of Vector-Borne Diseases (DVBD) at Fort Collins, Colorado, which performs *F. tularensis* testing. It was decided by the CDC components that each respective division lacked the other’s proficiency to test the samples for both pathogens, and that since the sample wasn’t confirmed by culture, the FADL could handle the sample under BSL-2 conditions. Subsequently, the FADL, upon guidance from the former Commander (COL von Tersch) of the U.S. Army PHC-C, did not to test the animal for rabies, because of the potential for aerosolization of the organism to themselves during rabies testing (cutting the skull to obtain brain tissue). Under aerosol conditions,* F. tularensis* is more infectious, which would mean that this organism would need to be handled under BSL-3, which deals with high risks related to containment and requires the appropriate use of personal protective measures including the use of a suitable biosafety cabinet and/or an N100 half-face respirator during any potential aerosolizing procedures such as centrifugation or vortexing. The FADL is only a BSL-2 laboratory. Therefore, a conclusion could not be made as to whether the cat was negative or positive for rabies. The Commander did allow for small tissue samples to be excised from the cat head to be sent to USAMRIID (a BSL-4 laboratory) per their request. At this point, the cat head and samples were packaged according to established internal SOPs for potentially hazardous materials, placed in a refrigerator, and the laboratory was decontaminated. The tissue samples were then sent to USAMRIID. All other involved material including the cat head was autoclaved twice and incinerated in the FADL’s hazardous waste stream.

Veterinarian staff and laboratory personnel must clinically manage esoteric, unexplained, or questionable pet death examination as if the animal were contaminated. It would be judicious for veterinarians to employ standard precautions (hand washing, gloves, mask, and eye protection when procedures are likely to generate splashes or sprays), and disinfect contaminated surfaces and equipment during examination of their animal patients and in autopsy analysis to mitigate the risk of exposure to tularemia. Because *F. tularensis* is challenging to grow and precarious to manage, laboratory personnel needs to be notified whenever tularemia is considered. According to the American Society for Microbiology, at a minimum, routine specimens may be processed in the clinical laboratory using BSL-2 precautions^35^. Any isolate suspected of being *F. tularensis* must be handled using BSL-3 standard precautions to prevent skin contamination and creation of aerosols when working with potentially infectious specimens or cultures of* F. tularensis* and necessitates immediate referral to the state public health laboratory or another appropriate laboratory facility (such as USAMRIID) for further evaluation^35^. Work has to be performed in a class II or III biohazard hood. It would be prudent for laboratory personnel to develop an SOP for known abnormal samples and increase communication with the customer as to the condition of the sample for possible preliminarily screening on various rapid diagnostic equipment. This case underscores the importance of using a sequence of biosafety measures for those working with tularemia, as there is often no discernible breach in laboratory protocol and only a low inoculum is required for infection. The challenging testing procedures by different laboratories to correlate our laboratory results also emphasize the point that this organism is fastidious and may not grow on some regularly used media.

Tularemia should be considered in the differential diagnosis in cats with signs of a febrile illness, with or without lymphadenopathy, malaise, and history of recent ingestion of wild prey. Other applicable risk factors to consider include tick exposure and contact with wild mammals, as well as whether the animal is found where the disease is endemic, which is North America and throughout Europe and Asia^7^. Etiological differentials that were considered in this case included *Cytauxzoonosis*, and *Yersenia pseudotuberculosis* because of some similarities in the pathological profiles produced. In cats with *Cytauxzoonosis*, the spleen is typically enlarged, and congested, as our patient’s spleen was, but there are no white spots^41^. * Y. pseudotuberculosis* infection in cats histologically display yellowish-white nodules consisting of varying-sized foci of caseation necrosis, which was seen in our patient, and can be difficult to differentiate grossly from tularemia^41^. Due to the high risk of transmission of *F. tularensis* through a bite, the prevalence of the organism in central Kansas, and the gross appearance of the spleen, this pathogen was suspected and identified in this case.

We recommend that disease reporting requirements in Kansas and other states make 'zoonotic diseases of public health significance ' reportable by veterinarians to the state health departments, giving veterinary professionals authorization to report a comprehensive range of disease cases based on situational risk. This recommendation can lead to a partnership and collaboration that state health departments and animal agencies can appreciate and be instrumental in disease prevention efforts. Good communication and collaboration between civilian and military pathologists, state health department epidemiologists, civilian and military veterinarians, civilian and Department of Defense laboratorians, and civilian and military primary and emergency department healthcare providers are vital and can reduce time to clinical diagnosis and treatment of the patient (animal and human). After all, tularemia can be transmitted to pets and humans with implacable consequences, if not treated early. Also, client education on tularemia for not only owners, but also clinical veterinarians, along with taking precautions to prevent it, can significantly minimize the risks of exposure to both pets and humans. Our case study is a reminder for civilian and military human and animal health professionals to work together to recognize, prevent, and control zoonotic diseases such as tularemia.

**Impacts**

- A case report of presumptive F. tularensis and possible rabies exposure transmission from a pet cat to its owner demonstrates the obligation for cooperation between animal health, human health, and public health professionals in the management of zoonotic diseases.
- In regards to household pets, cats are more susceptible to tularemia than dogs.
- Virtually all of the documented cases of tularemia in domestic cats and those involving transmission of this disease from cats to humans have these associated factors: these cats are permitted to roam outside of the home in rural or near rural areas; these cats hunt, kill, and consume infected small rodents and rabbits.
- Rabbits and wild rodent ingestion are probably the most common mode of infection in household pets seen by veterinary medicine, but ticks and other vectors can transmit.
- Tularemia should be considered in the differential diagnosis in cats with signs of a febrile illness, with or without lymphadenopathy, malaise, and history of recent ingestion of wild prey.
- F. tularensis has been identified as a category A bioterrorism agent by the Centers for Disease Control and Prevention. A weapon using airborne F. tularensis would most likely result in an outbreak of inhalational tularemia three to five days later, marked by an acute, undifferentiated febrile illness with predominant manifestations of pneumonia, pleuritis, and hilar lymphadenopathy. Clustered cases occurring without the expected epidemiologic exposures to animals, insects, or environmental activities should indicate the probability of a bioterrorism event.
- A very small dose of F. tularensis is infectious; the bacteria is zoonotic via vector or aerosol or accidental inoculation, so PPE is extremely important for laboratory personnel.
- F. tularensis is difficult to grow and dangerous to handle; therefore, laboratory personnel should be notified whenever tularemia is considered or suspected.
- While most laboratories assume an inherent risk through the course of normal testing, laboratories should develop an SOP for known abnormal samples.

## Funding Information

The authors acknowledge that there is no grant support or other funding sources for this PLoS Currents Outbreaks case report.

## Competing Interests

Dr. Ralph A. Stidham, on behalf of all the authors of the manuscript submitted to PLoS Current Outbreaks DECLARE that no competing interests exist.

## Data Availability

We did have approval to reproduce tables, "Zoonoses cats" and "Zoonoses cats, continued," by UpToDate, in your PLOS Current manuscript. Unfortunately, UpTodate cannot allow these tables to be reprinted under the Creative Commons Attribution (CC BY) 4.0 license, according to Mr. Jason Davis, UpToDate Journal Rights manager. He informed us that they understood that the article will be published under the CC BY and they had no objection to this use so long as the tables are included within the context of the article. However, they cannot allow the tables to be individually reprinted under the CC BY because UpToDate does not use this publishing model. So, we simply referenced the tables (in reference #3) without putting the tables(s) in the article.

## Corresponding Author

Address correspondence to Dr. Stidham at Ralph.a.stidham.civ@mail.mil

## Additional Author Information

†Dr. Freeman is presently a postdoctoral associate at Koch Institute for Integrative Cancer Research at Massachusetts Institute of Technology (MIT), Cambridge, Massachusetts, United States of America.

†COL von Tersch is presently the Assistant Deputy Chief of Staff (Public Health - Labs), Office of the Surgeon General / Director, Laboratory Sciences Directorate, Army Public Health Center, Aberdeen Proving Ground, Maryland, United States of America

## References

[1] Dennis DT, Inglesby TV, Henderson DA, Bartlett JG, Ascher MS, Eitzen E, Fine AD, Friedlander AM, Hauer J, Layton M, Lillibridge SR, McDade JE, Osterholm MT, O'Toole T, Parker G, Perl TM, Russell PK, Tonat K. Tularemia as a biological weapon: medical and public health management. JAMA. 2001 Jun 6;285(21):2763-73. PubMed PMID:11386933. 11386933 10.1001/jama.285.21.2763

[2] Overgaauw PA, van Zutphen L, Hoek D, Yaya FO, Roelfsema J, Pinelli E, et al. Zoonotic parasites in fecal samples and fur from dogs and cats in the Netherlands. Vet Parasitol. 2009;163:115–22. DOI: 10.1016/j.vetpar.2009.03.044. 10.1016/j.vetpar.2009.03.044 19398275

[3] Kotton CN. Zoonoses from cats. In: UpToDate, Post TW (Ed), UpToDate, Waltham, MA. (Accessed on January 7, 2017). Copyright © 2017 UpToDate, Inc.

[4] Pickering LK, ed. Tularemia. In: Red Book: 2009 Report of the Committee on Infectious Diseases. 28th ed. Elk Grove Village, IL: American Academy of Pediatrics, 2003:666-667.

[5] Feldman, KA. Zoonosis Update: Tularemia. Vet Med Today, JAVMA; 2003; 222: 725-30. 12675294 10.2460/javma.2003.222.725

[6] Centers for Disease Control and Prevention. Tularemia-United States, 1990-2000. MMWR Morb Mortal Wkly Rep; 2002; 51: 9. Available at https://www.cdc.gov/mmwr/preview/mmwrhtml/mm5109a1.htm. Accessed January 7, 2017.

[7] Tarnvik A, Berglund L. Tularaemia. Eur Respir J 2003; 21: 361-73. Available at http://erj.ersjournals.com/content/21/2/361.short. Accessed 8 January 2017. 10.1183/09031936.03.00088903 12608453

[8] Larson, M. A., Fey, P. D., Hinrichs, S. H., & Iwen, P. C. (2014). Francisella tularensis Bacteria Associated with Feline Tularemia in the United States. Emerging Infectious Diseases, 20(12), 2068–2071. http://doi.org/10.3201/eid2012.131101 10.3201/eid2012.131101 PMC4257795 25424732

[9] Feldman K, Enscore R, Lathrop S et al. An outbreak of primary pneumonic tularemia on Martha's Vineyard N Engl J Med 2001; 345: 1601-6. Available at http://www.nejm.org/doi/full/10.1056/NEJMoa011374. Accessed January 5, 2017. 10.1056/NEJMoa011374 11757506

[10] Arav-Boger R. Cat-bite tularemia in a seventeen-year-old girl treated with ciprofloxacin. Pediatr Infect Dis J 2000;19:583–584. 10.1097/00006454-200006000-00024 10877184

[11] Liles WC, Burger RJ. Tularemia from domestic cats. West J Med 1993;158:619–622. 8337864 PMC1311795

[12] Capellan J, Fong IW. Tularemia from a cat bite: case report and review of feline-associated tularemia. Clin Infect Dis 1993;16:472–475. 10.1093/clind/16.4.472 8513049

[13] Centers for Disease Control and Prevention. Tularemia associated with domestic cats—Georgia, New Mexico. MMWR Morb Mortal Wkly Rep 1982;31:39–41. 6799767

[14] Evans ME, McGee ZA, Hunter PT, et al. Tularemia and the tomcat. JAMA 1981; 246:1343. 7196465

[15] Gallivan MV, Davis WA II, Garagusi VF, et al. Fatal-cat transmitted tularemia: demonstration of the organism in tissue. South Med J 1980;73:240–242. 6153473

[16] Magnarelli, L., S. Levy, and R. Koski, 2007: Detection of antibodies to Francisella tularensis in cats. Res. Vet. Sci. 82(1); 22–26. 16914176 10.1016/j.rvsc.2006.06.003

[17] Centers for Disease Control and Prevention (CDC). Tularemia - United States, 2001-2010. MMWR Morb Mortal Wkly Rep 2013; 62:963. Available at https://www.cdc.gov/mmwr/preview/mmwrhtml/mm6247a5.htm. Accessed January 6, 2017. PMC4585636 24280916

[18] World Health Organization. WHO guidelines on tularaemia. Geneva: World Health Organization; 2007;7. Available at http://www.who.int/csr/resources/publications/deliberate/WHO_CDS_EPR_2007_7/en/. Accessed January 13, 2017.

[19] Adams D, Fullerton K, Jajosky R, et al. Summary of Notifiable Infectious Diseases and Conditions - United States, 2013. MMWR Morb Mortal Wkly Rep 2015; 62:1. Available at https://www.cdc.gov/mmwr/preview/mmwrhtml/mm6253a1.htm. Accessed January 15, 2017. 10.15585/mmwr.mm6253a1 26492038

[20] Centers for Disease Control and Prevention (CDC). Increase in human cases of tularemia – Colorado, Nebraska, South Dakota, and Wyoming, January-September 2015, MMWR Morb Mortal Wkly Rep 2015; 47:1317-1318. Available at https://www.cdc.gov/mmwr/preview/mmwrhtml/mm6447a4.htm. Accessed January 14, 2017. 10.15585/mmwr.mm6447a4 26632662

[21] Friend, M. Tularemia: Reston, Va., U.S. Geological Survey, Circular 1297; 2006:11. Available at https://pubs.usgs.gov/circ/1297/report.pdf. Accessed December 28, 2017.

[22] Hornick R. Tularemia revisited. N Engl J Med 2001; 345:1637–9. Available at http://www.nejm.org/doi/full/10.1056/NEJM200111293452211. Accessed December 30, 2017. 10.1056/NEJM200111293452211 11757513

[23] Mani RJ, Morton RJ, Clinkenbeard KD. Ecology of tularemia in Central US Endemic Region. Current Tropical Medicine Reports. 2016 Sep 1;3(3):75-9. 10.1007/s40475-016-0075-1 PMC4967422 27525215

[24] Jellison, W.L., Tularemia in North America 1930-1974: Missoula, Montana, University of Montana, 1974; 276 p.

[25] Sjöstedt A., Francisella. In: Garrity G et al., eds. The Proteobacteria, Part B, Bergey’s Manual of Systematic Bacteriology, New York, NY, Springer, 2005;200–210.

[26] World Health Organization. Health aspects of chemical and biological weapons. Geneva, Switzerland: World Health Organization; 1970: 75-76. Available at http://apps.who.int/iris/bitstream/10665/39444/1/24039.pdf. Accessed January 5, 2017.

[27] Centers for Disease Control and Prevention. Biological and chemical terrorism: strategic plan for preparedness and response: recommendations of the CDC Strategic Planning Workgroup. MMWR Morb Mortal Wkly Rep; 2000;49 (RR-4): 1-14. Available at https://www.cdc.gov/mmwr/preview/mmwrhtml/rr4904a1.htm. Accessed January 7, 2017. 10803503

[28] Pohanka M, Skládal P. Bacillus anthracis, Francisella tularensis and Yersinia pestis. The most important bacterial warfare agents - review. Folia Microbiol (Praha). 2009. 54(4):263-72. 10.1007/s12223-009-0046-1 19826916

[29] Centers for Disease Control and Prevention. 2016 Nationally notifiable conditions. National notifiable diseases surveillance system (NNDSS). Available at https://wwwn.cdc.gov/nndss/conditions/notifiable/2016/. Accessed January15, 2017.

[30] Armed Forces Health Surveillance Center: Armed Forces Tri-Service Reportable Events Guidelines & Case Definitions, March 2012. Available at http://www.afhsc.mil/documents/pubs/documents/TriService_CaseDefDocs/Armed ForcesGuidlinesFinal14Mar12.pdf; accessed January 7, 2017.

[31] Pike, R. M. 1976. Laboratory-associated infections: summary and analysis of 3921 cases. Health Lab. Sci. 13:105-114. 946794

[32] Rusnak JM , Kortepeter MG , Hawley RJ , Anderson AO , Boudreau E , Eitzen E. Risk of occupationally-acquired illnesses from biological threat agents in unvaccinated laboratory workers. Biosecurity Bioterrorism. 2004; 2:281–293. 10.1089/bsp.2004.2.281 15650438

[33] US Department of Health and Human Services. Centers for Disease Control and Prevention. National Institutes of Health. 2007. Biosafety in microbiological and biomedical laboratories (BMBL). 5th ed. US Printing Office, Washington, DC. Available at https://www.cdc.gov/biosafety/publications/bmbl5/; accessed January 21, 2017.

[34] American Society for Microbiology. Sentinel level clinical laboratory guidelines for suspected agents of bioterrorism and emerging infectious diseases: Francisella tularensis. Available at https://www.asm.org/images/PSAB/LRN/Tularemia316.pdf. Accessed January 5, 2017.

[35] Shapiro, D Schwartz DR. Exposure of laboratory workers to F. tularensis despite a bioterrorism procedure. J Clin Microbiol 2002; 40: 2278–81. 10.1128/JCM.40.6.2278-2281.2002 PMC130659 12037110

[36] Singh, K. 2009. Laboratory acquired infections. Clin. Infect. Dis. 49:142–7. 10.1086/599104 PMC7107998 19480580

[37] Sewell DL. Laboratory associated infections and biosafety. Clin Microbiol Rev 1995; 8:389–405. 10.1128/cmr.8.3.389 PMC174631 7553572

[38] Centers for Disease Control and Prevention Recognition of illness associated with the intentional release of a biologic agent. MMWR Morb Mortal Wkly Rep. 2001;50:893–7. Available at https://www.cdc.gov/mmwr/preview/mmwrhtml/mm5041a2.htm. Accessed January 8, 2017. 11686473

[39] Veterinary Infection Control Committee. National Association of State Public Health Veterinarians. Compendium for standard veterinary precautions. JAVMA. 2015; 1257-1260. Available at https://avmajournals.avma.org/doi/abs/10.2460/javma.247.11.1252. Accessed January 17, 2017. 10.2460/javma.247.11.1252 26594810

[40] Colorado State University, James L. Voss Veterinary Teaching Hospital. Infection Control and Biosecurity Standard Operating Procedures (SOP); 2015: 146-150;175. Available at https://avmajournals.avma.org/doi/abs/10.2460/javma.237.12.1403. Accessed February 15, 2017.

[41] Maxie G. The hematopoietic system. In: Maxie G. eds. Jubb, Kennedy, and Palmer’s Pathology of Domestic Animals. 2nd ed. Ontario: Saunders LTD, 2007; 2786-2793.

